# Supplementary material for: Antibiotic Exposure, Common Morbidities and Main Intestinal Microbial Groups in Very Preterm Neonates: A Pilot Study
Source: Antibiotics (Basel). 2022 Feb 12;11(2):237. doi: 10.3390/antibiotics11020237 (PMC8868158; doi:10.3390/antibiotics11020237)
Supplement: Supplementary file 1 [file antibiotics-11-00237-s001.zip › antibiotics-1544902-supplementary.pdf]

Table S1. Changes of bacterial counts at each time point following a multiple linear regression analysis.

Variables	T0 (within 48 hours of life)	T1 (15 days of life)	T2 (30 days of life)	T3 (90 days of life)
Days of hospital stay	*Clostridium* cluster I	NS	*B. fragilis* group	NS
Beta	1.75		-0.69	
SE	0.008		0.01	
p-value	0.002		0.002	
R^2	0.039		-0.039	
Days on mechanical ventilation	*Clostridium* cluster I	NS	*B. fragilis* group	NS
Beta	-1.26		-0.62	
SE	0.021		0.02	
p-value	0.007		0.009	
R^2	-0.084		0.087	
Days on parenteral nutrition	NS	NS	*B. fragilis* group	NS
Beta			0.71	
SE			0.026	
p-value			0.006	
R^2			0.087	
Days at full enteral feeding	NS	NS	NS	NS
Days at the beginning of enteral feeding	NS	NS	NS	NS
Total days on antibiotics (from 0 to 15 days of life)	NS	NS	NS	NS
Total days on antibiotics (from 0 to 30 days of life)	NS	NS	NS	NS
Total days on antibiotics (from 0 to 90 days of life)	NS	NS	NS	NS

Beta, Standardized coefficient; SE, Standard Error as unstandardized coefficient.

NS, not significant
